# 151. Simplifying Empiric Antimicrobial Therapy Selection for Lower Respiratory Tract Infections in Intensive Care Unit Patients: Using Resistance Frequency to Guide Decision Making

**DOI:** 10.1093/ofid/ofab466.151

**Published:** 2021-12-04

**Authors:** Kenneth Klinker, Levita K Hidayat, C Andrew DeRyke, Mary Motyl, Karri A Bauer

**Affiliations:** 1 Merck & Co, Inc, Kenilworth, New Jersey; 2 Merck & Co, San Clemente, California; 3 Merck & Co., Inc., Kenilworth, New Jersey

## Abstract

**Background:**

In the US, the burden of multidrug resistant bacterial infections, including carbapenem-resistant *P. aeruginosa* (CRPA) and ESBL-producing *Enterobacterales* (ESBL-E), is substantial. These resistant pathogens may affect the delivery of timely effective therapy. The aim of this study is to evaluate beta-lactam (BL) susceptibility trends based on the aggregate frequency of CRPA and a combined ESBL-E phenotype (*K. pneumoniae* (KPn) + *E. coli* (EC)) observed in critically ill patients with lower respiratory tract infections (LRTI).

**Methods:**

In 2016-2019, ~20 US institutions per year submitted up to 250 gram-negative pathogens as part of the Study for Monitoring Antimicrobial Resistance Trends. A total of 871 PA, 380 KPn, and 336 EC isolates were collected from ICU patients with LRTI. MICs were determined using broth microdilution and interpreted using 2021 CLSI breakpoints. ESBL-E phenotype was defined as: ceftriaxone MIC ≥ 2 mcg/mL. Institutions were stratified into two groups based on frequency of CRPA and combined ESBL-E phenotype: Group 1: CRPA ≤ 15% and ESBL-E ≤ 15%; Group 2: CRPA > 15% and ESBL-E > 15%. Based on CLSI guidance, an empiric antibiotic susceptibility threshold of ≥90% was deemed optimal.

**Results:**

Overall, CRPA and ESBL-E phenotypes were identified in 28.4% and 21.2% of isolates, respectively. Aggregate BL susceptibility in group 1 was above the 90% threshold for cefepime (FEP), piperacillin/tazobactam (TZP), meropenem (MEM), ceftolozane/tazobactam (C/T), and imipenem/relebactam (I/R) (Table 1). However, as frequency of CRPA and ESBL-E exceeded 15%, aggregate BL susceptibility declined to 77.3%, 79.3%, and 86.2% for FEP, TZP, and MEM, respectively. In contrast, C/T and I/R maintain susceptibility above the empiric susceptibility threshold.

Table 1. Aggregate susceptibility of P. aeruginosa, E. coli, and K. pneumoniae ICU LRTI isolates stratified by resistance frequency: Best- (Group 1) and worst-case (Group 2) scenarios

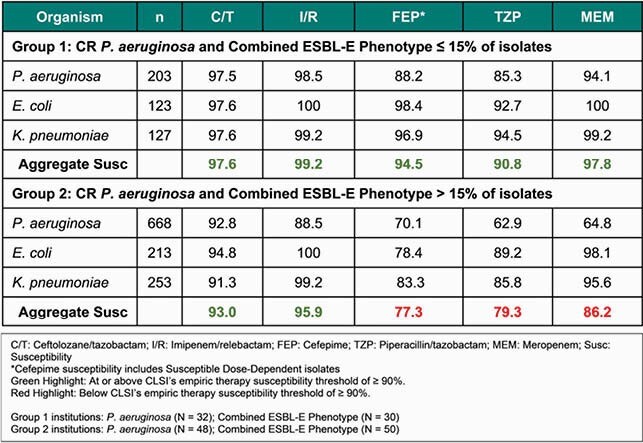

**Conclusion:**

In ICU patients, exceeding CRPA and combined ESBL-E phenotype frequency of 15% for both classifications, impacts susceptibility to 1^st^ line BL’s resulting in a failure to achieve empiric susceptibility thresholds. This stratification could serve as a decision point for triggering earlier susceptibility testing or modifying empiric therapy recommendations for LRTI to include newer agents pending microbiology results.

**Disclosures:**

**Kenneth Klinker, PharmD**, **Merck & Co., Inc.** (Employee, Shareholder) **Levita K. Hidayat, PharmD BCIDP**, **Merck & Co., Inc.** (Employee, Shareholder) **C. Andrew DeRyke, PharmD**, **Merck & Co., Inc.** (Employee, Shareholder) **Mary Motyl, PhD**, **Merck & Co., Inc.** (Employee, Shareholder) **Karri A. Bauer, PharmD**, **Merck & Co., Inc.** (Employee, Shareholder)

